# Achieving inter- and transdisciplinarity in Ecohealth: insights from a rodent-borne disease project in a polycrisis era

**DOI:** 10.3389/fvets.2024.1235183

**Published:** 2024-12-18

**Authors:** Isabelle Arpin, Clémence Massart, Vincent Bourret, Guillaume Castel, Valeria Carolina Colombo, Jana Eccard, Jasmin Firozpoor, Maciej Grzybek, Heikki A. Henttonen, Herwig Leirs, Andrew McManus, Ben Roche, Tarja Sironen, Vincent Sluydts, Peter Stuart, Annetta Zintl, Nathalie Charbonnel

**Affiliations:** ^1^Institut national de recherche pour l’agriculture, l’alimentation et l’environnement (INRAE), Grenoble, France; ^2^Comportement et Ecologie de la Faune Sauvage (CEFS), Institut national de recherche pour l’agriculture, l’alimentation et l’environnement (INRAE), Toulouse, France; ^3^Centre de Biologie pour la Gestion des Populations (CBGP), Institut national de recherche pour l’agriculture, l’alimentation et l’environnement (INRAE), Montpellier, France; ^4^University of Antwerp, Antwerp, Belgium; ^5^University of Potsdam, Potsdam, Germany; ^6^Medical University of Gdańsk, Gdansk, Poland; ^7^Natural Resources Institute Finland (Luke), Helsinki, Finland; ^8^Munster Technological University, Cork, Ireland; ^9^UMR5290 Maladies Infectieuses et Vecteurs: Ecologie, Génétique, Evolution et Contrôle (MIVEGEC), Montpellier, France; ^10^University of Helsinki, Helsinki, Finland; ^11^University College Dublin, Dublin, Ireland

**Keywords:** wicked problems, wickedness, interdisciplinary research, transdisciplinary research, Ecohealth, rodent-borne diseases, polycrisis era

## Abstract

**Introduction:**

Inter- and transdisciplinary research (ITDR) is increasingly promoted to address “wicked problems”, particularly in health sectors adopting approaches like Ecohealth. Our Ecohealth-inspired project on rodent-borne diseases, initiated just before the COVID-19 pandemic, provided an opportunity to evaluate ITDR implementation.

**Methods:**

We employed a recently developed semi-quantitative evaluation method to measure our project’s success in achieving ITDR and analyzed factors influencing this achievement.

**Results:**

The project showed strengths in system description, team task allocation, and data sharing, but had lower scores in engaging societal actors throughout the project cycle.

**Discussion:**

We identified the underexplored influence of problem wickedness as a critical determinant of ITDR success. Addressing rodent-borne diseases, a less wicked problem, limited engagement potential but enabled constructive dialog with local actors. These insights are vital for addressing variably wicked problems in a polycrisis era. We propose recommendations to strengthen researchers’ capacities, particularly in Ecohealth.

## 1 Introduction

In recent decades, the term ‘wicked problems’ has gained prominence to describe issues marked by high levels of uncertainty and a lack of consensus on both definitions and solutions (1–3). Emerging zoonotic diseases, with their complexity and unpredictability, exemplify such problems (4–7). Wicked problems vary in degree based on two main criteria: cognitive complexity—reflecting the challenges in defining the problem and identifying solutions—and political complexity, which relates to the diversity and conflict among stakeholders affected by or invested in the issue (1, 8). The notion of ‘super wicked problems’, which includes the added urgency of resolution, underscores the escalating need for effective approaches in the face of crises like COVID-19 (9, 10).

Traditional disciplinary research struggles to address these complex issues. Interdisciplinary and transdisciplinary research (ITDR) has emerged as a promising approach, particularly suited not to solve but to better address wicked problems by incorporating diverse perspectives. ITDR’s integrative potential has shown promise in understanding zoonotic diseases (6, 11). Interdisciplinarity merges insights, data, and methodologies from multiple disciplines (12), while transdisciplinarity extends beyond academic boundaries to include societal actors, aiming to produce actionable insights into ‘real-world’ problems (13, 14). Thus, ITDR not only enhances understanding but also seeks to improve conditions through collaborative, knowledge-based interventions.

The relevance of ITDR to current societal challenges is largely accepted. However, achieving it remains challenging, especially given the predominantly disciplinary structure of academic institutions (13, 15). Multiple factors—including individual, institutional, geographical, and project-specific—can influence the success of ITDR initiatives (16). This article contributes to understanding the factors influencing ITDR through a case study of a project on rodent-borne diseases (RBDs), an area of zoonotic disease research with both high cognitive complexity and relatively lower political complexity in Europe.

Rodents, as a diverse and highly adaptable group of mammals, have historically played a pivotal role in the transmission of zoonotic agents, such as bacteria (e.g., *Leptospira*, *Borrelia*), viruses (e.g., hantaviruses), and protozoa (e.g., *Toxoplasma gondii*), often with significant health implications (17, 18). While RBDs are complex in terms of human-rodent-pathogen interactions—characterized by indirect transmission and frequently nonspecific symptoms—they typically involve fewer societal actors and conflicts than other zoonotic diseases such as avian influenza or SARS. Consequently, RBDs can be considered to be ‘weakly wicked’ problems in Europe.

Despite the recognized importance of ITDR in addressing zoonotic diseases, RBD-focused research has seen limited adoption of this approach. While initiatives such as the European EDEN and EDENext projects promoted the One Health approach in the RBD research community (19), few RBD studies have embraced ITDR in a comprehensive way (exceptions include (20, 21)). By analysing an RBD project launched just before the COVID-19 pandemic in early 2020, this article explores key factors that influence the capacity to achieve inter- and transdisciplinarity in research on emerging zoonotic diseases and discusses the implications of such approaches in navigating a ‘polycrisis era’ (22).

## 2 Materials and methods

This section presents an overview of our project, followed by the method we used to evaluate its level of interdisciplinarity and transdisciplinarity.

### 2.1 A European project on rodents, humans, and pathogens

**Figure 1:**
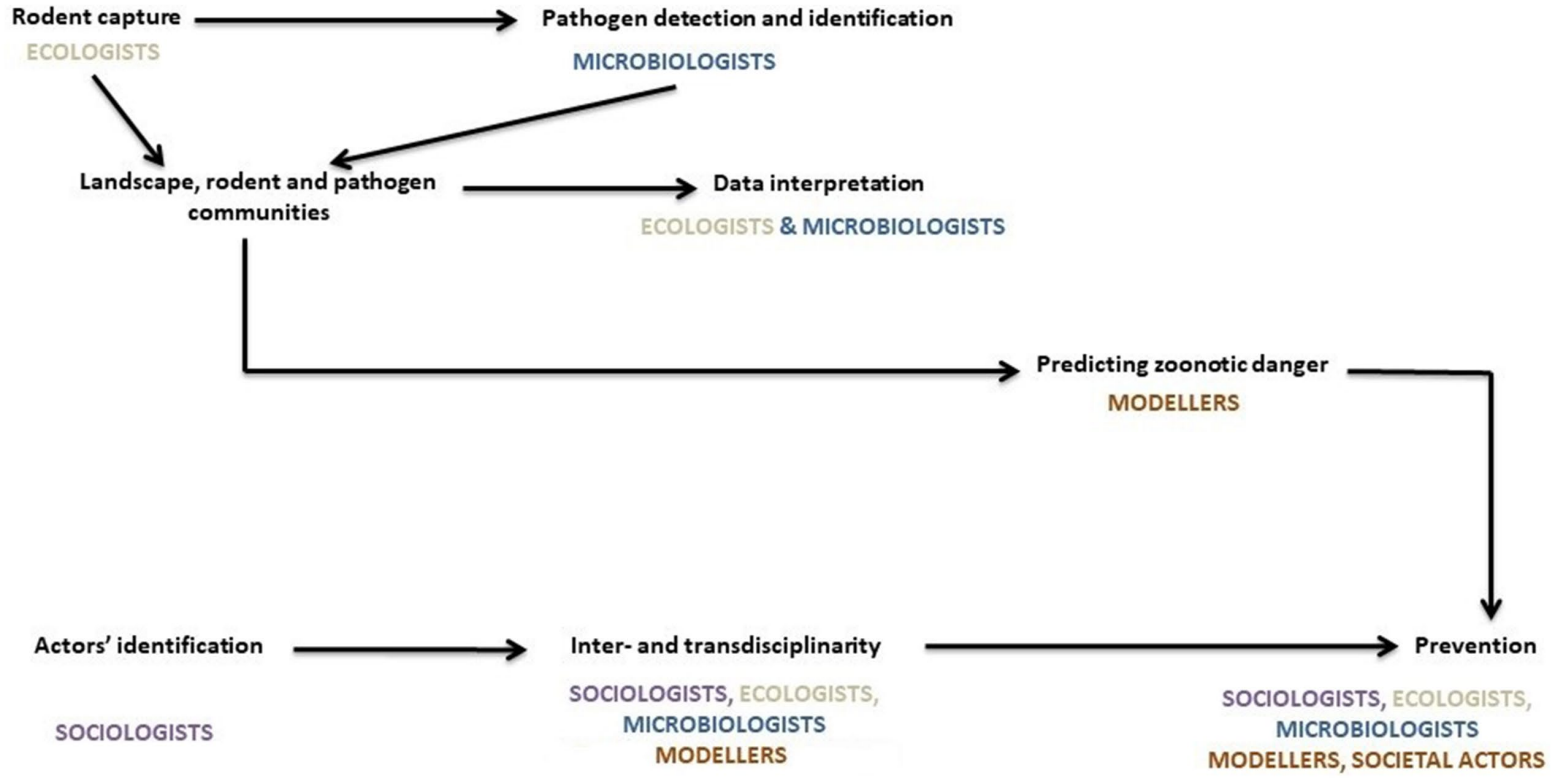
Distribution of tasks between members during the course of the project.

**Figure 2:**
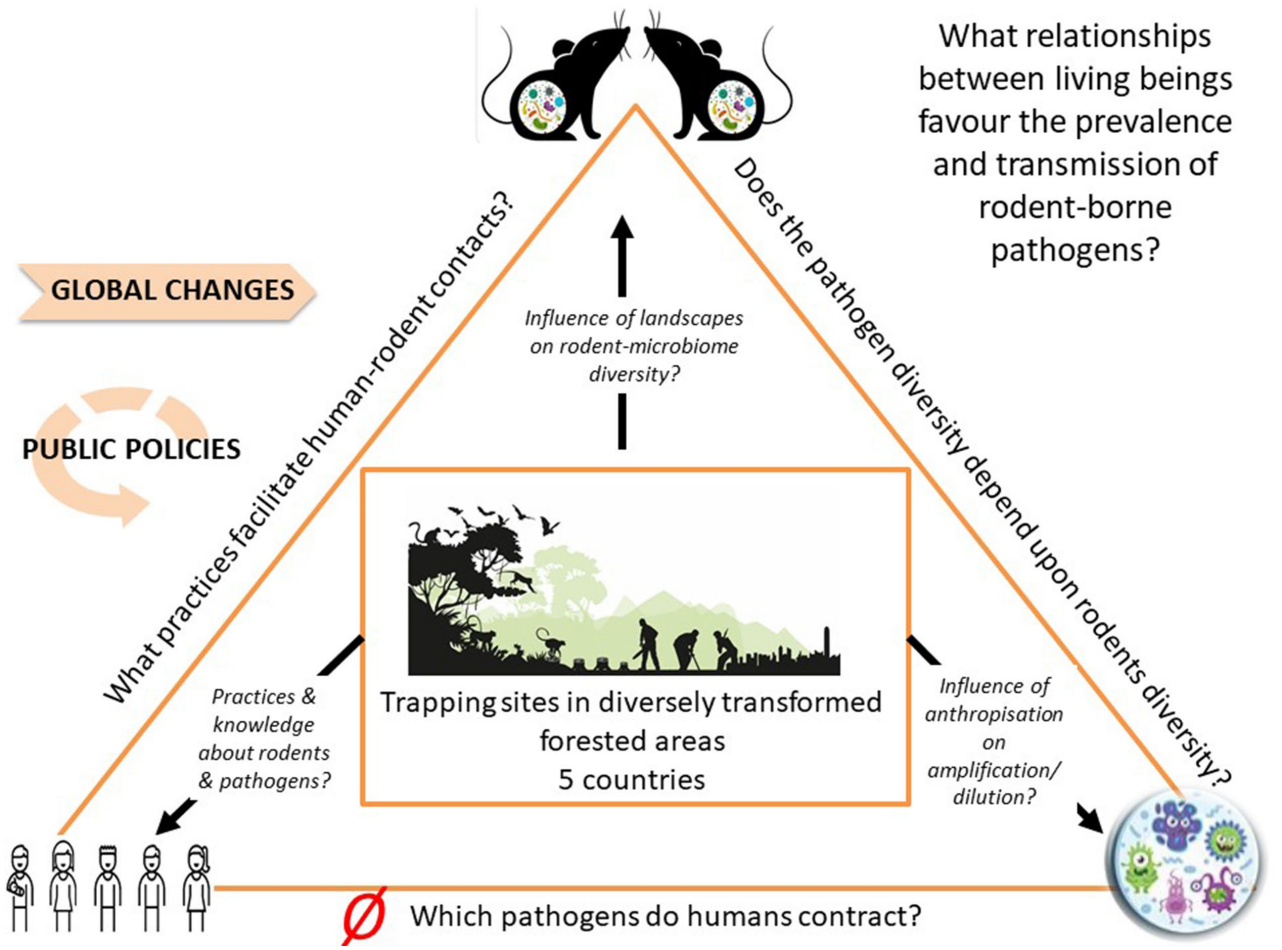
Research framework for the project. This diagram illustrates the interconnected elements of our project. The central rectangle represents the project’s field sites, while the two rodents at the top symbolize the diverse rodent species found within these sites and their gut microbiomes. The circle in the lower right indicates the variety of pathogens present in these rodents. The figures on the lower left represent the site managers and users. Research questions are positioned along the edges of the triangle, with a red Ø symbol marking that not explored within our project.

Our initial focus was predominantly on rodent-pathogen dynamics, rather than RBDs directly. However, the need for funding pushed us toward broadening our objectives and incorporating public health concerns. We therefore designed an interdisciplinary and transdisciplinary framework to address the interconnected biological and social factors influencing zoonotic disease dynamics. Our project aimed to (i) elucidate the complex relationships within a multispecies community including rodents, humans, and pathogens; (ii) forecast how biodiversity changes may impact the epidemiology and emergence of RBDs; (iii) provide data on the distribution of rodents and rodent-borne diseases across Europe; and finally, (iv) inform public health campaigns on RBD prevention (see Figures 1, 2).

Our team was led by a French evolutionary ecologist with extensive experience in host-pathogen relationships. It comprised 25 scientists from six European countries, including ecologists, epidemiologists, virologists, modelers, two sociologists, but no human or animal health practitioners. Many team members had collaborated previously in European RBD-related projects, fostering strong interpersonal connections during annual week-long meetings in remote settings. In contrast, sociologists joined at a later stage. Moreover, the team included 11 short-term researchers (PhD students, postdocs, and research assistants) with no inter- and transdisciplinary experience, except for the junior sociologist. Notably, France was the only country where all disciplinary areas were represented.

The project utilized a site-based approach, studying rodent-human-pathogen interactions across 29 field sites in five countries (France, Belgium, Germany, Ireland and Poland). These sites included managed and protected forests, urban parks, and zoos, representing a range of environments where humans and rodents interact. A standardized protocol for rodent trapping and data collection was implemented over 2 years, capturing seasonal variations in rodent populations and analysing their pathogens and gut microbiomes. This allowed for comparative analysis of ecological and epidemiological data across different geographic and social contexts.

To strengthen transdisciplinary engagement, we planned to involve societal actors at both local and national levels in assessing knowledge and practices concerning rodents and RBDs. At the local level, we aimed to engage site managers (e.g., local services of the national forest service, urban park departments) and local physicians, while at the national level, we intended to include agencies and government departments overseeing public health and biodiversity conservation. We prepared for semi-structured interviews and participatory workshops with site managers and national representatives to understand their perspectives, needs, and existing practices. Additionally, two targeted questionnaires were developed: one for local site users and another for nearby physicians, gathering insights into public awareness and preventive actions around RBDs.

Monitoring of inter- and trandisciplinarity was integral to the project’s design to assess and facilitate collaboration. This included identifying challenges and developing solutions to improve inter- and transdisciplinary integration throughout the project’s duration.

A significant feature of our project was its timing, coinciding with the COVID-19 pandemic. In April 2020, the European Commission sought biodiversity and zoonosis-focused projects to address coronavirus risks. We applied for and received a ‘coronavirus extension,’ officially granted in September 2020, with fewer requirements in ITDR. In particular, this extension did not involve sociologists.

### 2.2 Evaluation method of ITDR

To evaluate the project’s level of inter- and transdisciplinarity, we applied the semi-quantitative EVOLvINC (Evaluating knOwledge Integration Capacity in multi-stakeholder governance) method (23). This method was selected based on: (1) its development by a consortium of health specialists and transdisciplinary research, ensuring relevance on One Health research (16, 23); (2) its semi-quantitative dimension, which enhanced credibility among life scientists; (3) its self-administered format, allowing iterative application throughout the project lifecycle (16).

EVOLvINC distinguishes three types of knowledge relevant to project stages: target knowledge (normative knowledge regarding desired future states); transformation knowledge (prescriptive knowledge for achieving these states), and systems knowledge (descriptive knowledge about the current state of the system). These knowledge types correspond to the formulation, implementation, and evaluation stages of the project cycle. EVOLvINC further breaks down these stages into six critical components: thinking and planning for project formulation, organization and working for implementation, and sharing and learning for evaluation. It provides a structured set of criteria and indicators to evaluate each component, employing a questionnaire with four-level Likert scales and a semi-quantitative algorithm to score and aggregate responses, visualisable through a spider diagram.

The implementation of the EVOLvINC method unfolded in four key steps:

1. Initial empirical study: the junior sociologist conducted interviews with all team members (June–September 2020), guided by a standardized interview format. Questions explored team members’ backgrounds, research interests, project motivations, success criteria, anticipated challenges, and expectations regarding ITDR. All interviews were recorded, and summaries were shared with participants for verification. The senior sociologist also interviewed the junior sociologist. In addition, the sociologists participated in all project meetings and presented their intermediary results to the rest of the team, in order to discuss challenges and potential improvements to ITDR practices within the project.
2. Questionnaire administration: the senior sociologist and the project coordinator independently completed the EVOLvINC questionnaire, drawing upon their respective knowledge of the project. Where scores varied significantly across countries or in levels of inter- and transdisciplinary collaboration, an average score was assigned.
3. Consensus building: the senior sociologist and the project coordinator compared their responses to reach a consensus on Likert-scale scores, addressing any discrepancies through discussion.
4. Team involvement: the final exercise involved an online meeting with the entire team to ensure that scores accurately reflected the collective perspective of the project, mitigating potential biases.

## 3 Results

This section presents the findings on the level of ITDR within our project. First, we analyse scores obtained using the EVOLvINC evaluation method, then identify key factors influencing the observed levels of interdisciplinarity and transdisciplinarity.

### 3.1 ITDR evaluation scores

**Figure 3:**
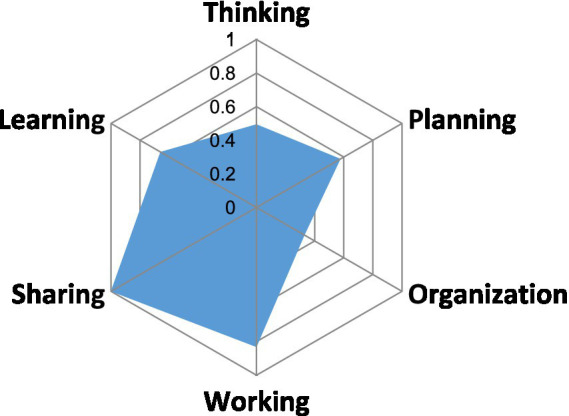
Spider diagramme showing the aggregate scores of our project for the six aspects identified in EVOLvINC.

The EVOLvINC method assessed our project against 22 criteria across the six key project stages, revealing strengths in data sharing and team dynamics, alongside challenges in stakeholder engagement and resource distribution (Table 1; Figure 3).

**Table 1:** Scores of our project for the six aspects identified in the EVOLvINC method (adapted from Hitziger et al. (23)).

Stages of project cycle	Aspect (score)	EVOLvINC criterion	Score of our project
Formulation	Thinking (0.5)	Inclusive design process	0.5
		Consideration of system features	1
		Leverage potential	0.33
	Planning (0.58)	Identification and engagement of sectors, actors, and stakeholders	0.66
		Reflexivity and adaptiveness	0.75
		Competences and methods	0.5
		Resource allocation	0.5
Implementation	Organization (0.33)	Internal team structure	1
		External stakeholder network	0.33
		Bridging knowledges	0.33
	Working (0.83)	Power distribution	0.67
		Leadership	1
		Conflict resolution	0.83
Evaluation	Sharing (1)	Processes for information exchange	0.83
		Data	1
		Methods and results	1
		Institutional memory	1
	Learning (0.66)	Individual learning	0.66
		Team learning	0.66
		Organisational learning	0.66
		Direct environment	0.33
		General environment	0.33

High scores (≥0.75) were achieved in nine criteria, particularly those related to data sharing. From the project’s outset, we established robust data-sharing protocols that ensured access to biological data for all team members and allowed for scheduled publication in open databases. Similarly, sociological data, including interviews and survey responses, were securely stored in the data portal of the sociologists’ institute and shared within the team. The high scores in these areas underscore the project’s commitment to transparency and internal knowledge flow, contributing substantially to EVOLvINC’s “Sharing” dimension. Our project also scored highly in criteria related to team structure, conflict resolution, reflexivity, and adaptiveness. Strong leadership and responsive coordination, especially during pandemic-related lockdowns, helped address challenges that could have hindered progress. For example, the creation of instructional videos on rodent dissection and pathogen identification enabled continuity in fieldwork and training during restricted periods. These elements not only enhanced team cohesion but also bolstered interdisciplinary collaboration, ensuring that the project’s objectives remained achievable despite external constraints.

Intermediate scores (0.5–0.75) were observed in eight criteria, reflecting partial success in achieving inclusivity in the design process, stakeholder engagement, resource allocation, competence development, and equitable power distribution. While the project brought together a range of disciplines, certain critical areas—especially in medical sciences, policy, and communication—were entirely missing. Limitations in human and financial resources impacted the sociological team’s contributions to the project, especially after the departure of the junior sociologist, which affected the project’s interdisciplinary scope and balance. Moreover, although team members gained valuable skills and refined their methods, these outcomes did not translate into significant paradigm shifts or alterations to the project’s underlying organization. Consequently, these intermediate scores reflected only moderate success in EVOLvINC’s “Learning” and “Planning” domains.

Low scores (<0.5) were recorded in five criteria, particularly in engaging societal actors—a shortcoming that weakened the project’s overall “Organization” score despite a well-functioning internal team structure. Notably, our attempts to engage local physicians were unsuccessful, as many were overextended by COVID-19-related demands. The distribution of questionnaires to site users yielded inconsistent responses across countries. In France, Ireland, and Belgium, we managed to engage some site users, either directly during rodent capture activities or by sending questionnaires through affiliated organizations, such as site management teams and sports organizations. However, the low response rate hindered our ability to perform robust statistical analyses. In Germany, no responses were gathered, and the questionnaire was not disseminated in Poland.

Interaction with site managers also varied: in France, longstanding partnerships facilitated active engagement, resulting in successful interviews and two participatory workshops that enriched our understanding of local perspectives on rodents and rodent-borne diseases. France also led in involving national societal actors across multiple sectors, further advancing the project’s transdisciplinary aims. However, limited involvement of societal actors during the project’s design phase restricted the extent of our transdisciplinary outcomes, particularly in fostering collaborative approaches from the outset. In contrast, interdisciplinary activities were more successful. Initial interviews with project members helped establish a shared understanding of research goals, and regular team meetings enabled interdisciplinary knowledge exchange, including reflective discussions on the complexities of rodent-borne diseases as a “wicked problem”.

### 3.2 Influencing factors

The level of inter- and transdisciplinarity achieved in our project was shaped by a range of facilitating and constraining factors across individual, institutional, project design, contextual, and problem wickedness dimensions (Table 2).

**Table 2:** Factors influencing inter- and transdisciplinarity in our project.

Category	Positive influence	Negative influence
Individual factors	Project coordinator’s commitment, relational and communication skills Openness of most project members to interdisciplinarity	Limited interdisciplinary and transdisciplinary experience among members
Institutional factors	Incentives to pursue inter- and transdisciplinary approaches	Lack of specific incentives for ITDR approaches on SARS-cov2 Time pressure in short-term projects Pressure to publish, limiting time for inter- and transdisciplinary activities
Project design and management	Site-based approach to studying relationships between rodents, pathogens, and humans	Lack of early involvement of societal actors in the project Lack of social scientists outside France High proportion of students with a disciplinary training
Geographical and linguistic factors		Physical distance between project members Use of English for interviews with societal actors at national level outside France, limiting engagement
Related to the pandemic	Increased awareness of zoonoses and their impact	Restrictions on in-person fieldwork and meetings during the first 2 years
Problem wickedness	Productive discussions with site managers on practical communication and management issues and on the health-biodiversity nexus	Difficulty engaging other societal actors

#### 3.2.1 Individual factors

The project coordinator’s commitment and expertise were pivotal in advancing interdisciplinary and transdisciplinary efforts. Her strong relational and communication skills helped bridge disciplinary divides and fostered connections with societal actors, particularly in France, where links were established with both local and national actors. Her leadership played a crucial role in maintaining team motivation, especially for the sociologists, who felt somewhat isolated and faced difficulties engaging societal actors. Team openness toward ITDR also supported interdisciplinary exchanges: for example, the two sociologists attended rodent biology sessions, while the life scientists actively participated in the sociological workshops. However, limited prior experience with transdisciplinary research initially caused misunderstandings. Life scientists, for example, initially viewed transdisciplinarity as simply communicating research findings to societal actors, rather than involving these actors integrally in the research design and implementation.

#### 3.2.2 Institutional factors

Institutional incentives had both positive and negative effects on ITDR. While ITDR approaches were encouraged in securing project funding, the dominant institutional focus on career advancement through disciplinary publications and the pressures of short-term project timelines created conflicting demands. These pressures resulted in an institutional pattern of promoting ITDR proposals without adequately supporting their ongoing interdisciplinary and transdisciplinary execution. Additionally, the COVID-19 pandemic altered institutional priorities, channeling resources toward traditional research on viral reservoirs rather than supporting ITDR practices.

#### 3.2.3 Project design and management

The design and management of the project presented challenges to achieving full transdisciplinarity. The absence of societal actors in the project’s early stages limited engagement opportunities, focusing discussions primarily on methodologies—such as site selection and rodent capture protocols—rather than on broader themes like the biodiversity-health nexus, which emerged only in France near the project’s end. Moreover, with no social scientists outside France, interdisciplinary and transdisciplinary integration in other countries was limited. The high proportion of students trained in specific disciplines also contributed to pressures to publish in their areas of expertise, leaving little time for ITDR. However, the site-based research approach facilitated engagement with local site managers, who showed interest in rodent pathogen data applicable to their areas, fostering experience-sharing among sites and with societal actors at national level.

#### 3.2.4 Contextual challenges

The COVID-19 pandemic heavily impacted the project, disrupting in-person fieldwork and forcing team and societal actor interactions into online formats during the first 2 years. Physical distance and language diversity further complicated communication and collaboration. While questionnaires were translated into multiple languages to enhance accessibility, the reliance on English in interviews and meetings often limited meaningful engagement with societal actors, particularly those outside of France.

#### 3.2.5 Problem wickedness

The relatively weak wickedness of RBDs influenced the project’s engagement dynamics. Although zoonoses attracted increased public interest due to COVID-19, RBDs were seen as less urgent compared to pandemic-related issues. This lower prioritization complicated efforts to engage societal actors, especially medical professionals, who were preoccupied with the pandemic. However, this lower wickedness also allowed for constructive dialog with site managers on prevention and rodent conservation without the heightened pressures associated with high-stakes zoonotic outbreaks. Additionally, the lower perceived wickedness of RBDs impacted funding access, as resources for pandemic-related research with fewer requirements for ITDR approaches became easily available.

## 4 Discussion

We begin by examining the advantages and limitations of the EVOLvINC method for assessing the level of inter- and transdisciplinarity (ITDR) within our project. Subsequently, we assess the level of ITDR achieved in our research on rodent-borne diseases (RBDs), considering various factors influencing these dynamics. Finally, we highlight a critical but underexplored factor—problem wickedness—emphasizing its influence on shaping ITDR levels and collaboration dynamics.

### 4.1 Limitations and advantages of the EVOLvINC method

The EVOLvINC method presented both strengths and limitations in evaluating ITDR in our project.

#### 4.1.1 Limitations

The method was relatively resource-intensive, requiring extensive input from both the sociologist and the project coordinator. The questionnaire included numerous items, some of which required in-depth reflection and consensus building (e.g., assessing the project’s leverage potential), which demanded significant time and coordination. Another limitation was the method’s limited capacity to distinguish between inter- and transdisciplinary interactions within certain criteria, such as the ‘Bridging knowledges’ criterion (23), which conflates efforts to connect knowledge across team members and stakeholders. This conflation obscured significant differences between inter- and transdisciplinary interactions—especially relevant in our case, where the two types of collaboration diverged notably. Additionally, aggregated scores masked the varying levels of ITDR achieved across the different countries involved in the project.

#### 4.1.2 Advantages

Despite these limitations, EVOLvINC offered substantial benefits. Its self-administrable nature enabled real-time assessments, fostering immediate project improvements rather than postponing adjustments to future projects. For instance, EVOLvINC results prompted us to organise a second participatory workshop in France, facilitating greater societal engagement during the project. Furthermore, EVOLvINC provided a structured, comprehensive and transparent framework for evaluating and discussing ITDR aspects, facilitating open dialog on sensitive issues such as conflict resolution. Beyond merely assessing scores, the method created a formalized space for collective reflection, where team members could collaboratively address the project’s inter- and transdisciplinary strengths and weaknesses. In summary, the advantages—such as promoting real-time feedback and collective discussions—significantly outweighed the limitations.

### 4.2 Level of inter- and transdisciplinarity and its influencing factors

#### 4.2.1 ITDR evaluation scores

The EVOLvINC analysis revealed that our project shared common characteristics with science-led initiatives, achieving high scores for all aspects related to the description of the system, the division of tasks within the team and the sharing of data and methods (16). However, the project exhibited comparatively low scores for integrating societal actors during all phases (problem formulation, implementation, and evaluation). According to Mobjörk’s (24) classification, our transdisciplinary engagement was more consultative than participatory, while the interdisciplinary component also remained constrained.

#### 4.2.2 Influencing factors

Many factors affecting ITDR in our project align with those documented in prior research. Established influences included individual motivations (25–27), institutional support (28), project design and management considerations (14, 29), geographical dispersion (30), and language barriers (31). Notably, the project coordinator’s commitment and skill, alongside the place-based research design, mitigated challenges such as the life sciences-social sciences imbalance and the limited ITDR experience among team members. However, an additional, less-recognized factor—problem wickedness—proved highly influential.

#### 4.2.3 Problem wickedness as a key factor in ITDR

In exploring factors that influenced ITDR, we found that the level of wickedness of the research problem has not received sufficient attention in the literature, despite its relevance to ITDR objectives. Typically, ITDR efforts are motivated by the need to address “wicked problems.” However, in the field of infectious diseases, few studies have explored the relationship between the level of problem wickedness and ITDR outcomes [but see (32–34)].

#### 4.2.4 Application to RBDs as a weakly wicked problem

RBDs in our study presented a moderately wicked problem, one less urgent than crisis-level zoonotic threats. This lower level of wickedness had a dual impact on ITDR. It created challenges in engaging certain societal actors, like physicians and site users, who perceived RBDs as low-priority issues, especially during the COVID-19 pandemic. However, the relatively lower wickedness facilitated productive collaborations with other stakeholders, such as site managers and national actors, enabling a focus on practical, site-specific concerns such as risk communication for staff, users, and the public. This moderate level of wickedness also allowed for calmer, more reflective discussions on the relationships between biodiversity conservation and public health, which may have been harder to achieve in a more urgent, high-stakes context.

#### 4.2.5 Absolute and relative wickedness

Our project also revealed the significance of both absolute and relative levels of wickedness. The COVID-19 pandemic—a “super wicked” problem—impacted our project in multiple ways, adding complexity by shifting some research efforts to coronavirus studies involving less inter- and transdisciplinary collaboration. This shift reflects the importance of viewing wicked problems as part of an interconnected “ecology of problems” (35), an idea resonant with the rising expression of a “polycrisis era” (22). Our findings underscore the need to consider both the relative and absolute wickedness of problems in ITDR.

In sum, our study highlights problem wickedness as an essential yet underrecognised factor in ITDR research. While established factors like leadership, team motivation and experience, institutional support, and project management significantly shaped our project, the relatively weak wickedness of RBDs influenced our ability to foster both interdisciplinary and transdisciplinary interactions. In particular, lower wickedness allowed for constructive site-based engagement but presented barriers to broader societal involvement. Further research is needed to explore how varying degrees of wickedness shape ITDR dynamics, particularly within One Health and emergent infectious disease contexts, to disentangle these effects from other well-known factors.

## 5 Conclusion

This study evaluated the inter- and transdisciplinary (ITDR) efforts within a One Health project on rodent-borne diseases (RBDs) using the EVOLvINC method, uncovering key factors that influenced its outcomes. While our findings reaffirm the value of well-established ITDR support mechanisms, they also highlight a critical but often-overlooked factor: problem wickedness. Specifically, we found that the weakly wicked nature of RBDs created both opportunities and limitations for ITDR, and this dynamic became particularly apparent when juxtaposed with the super-wicked problem of the COVID-19 pandemic. This insight holds pressing relevance in today’s polycrisis era, where societies must contend with a range of interwoven, complex problems, often without the capacity to foresee which seemingly manageable issues may escalate into crises. Recognizing and adapting to the degree of problem wickedness could therefore prove essential for the resilience of ITDR projects in this unpredictable landscape.

Our findings suggest operational implications that could strengthen ITDR projects, particularly for those addressing weakly wicked problems, and underscore the need to anticipate and adapt to different levels of problem wickedness:

- Encouraging reflexivity through continuous evaluation: employing a recognized evaluation tool like EVOLvINC fosters reflexivity about ITDR achievements and gaps, enabling teams to iteratively enhance inter- and transdisciplinary collaboration. Using such tools mid-project also supports adaptive management and the co-design of strategies to address emergent challenges.
- Assessing and adapting to problem wickedness: evaluating both the absolute and relative wickedness of a project’s focus is essential for shaping realistic engagement strategies. A nuanced understanding of problem wickedness can help anticipate challenges with societal engagement, funding access, and the shifting priorities of stakeholders, enabling projects to adjust approaches and maintain resilience amid change.
- Ensuring disciplinary balance and integrating social sciences: balanced disciplinary integration, especially involving social scientists as core team members, is critical for effective ITDR.
- Leveraging the value of place-based research: long-term, place-based research offers substantial benefits for fostering meaningful transdisciplinary connections. By grounding projects in local contexts, this approach cultivates stronger relationships with societal actors and deepens interdisciplinary understanding—essential in projects that address health and environmental challenges at the community level.

In this polycrisis era, as interconnected global challenges reshape the research landscape, these recommendations serve as a framework to enhance ITDR’s adaptability, relevance, and impact. Recognizing problem wickedness as a fundamental factor, alongside established ITDR supports, can empower researchers to better navigate the unpredictable evolution of today’s wicked problems.

## Data Availability

The raw data supporting the conclusions of this article will be made available by the authors, without undue reservation.
