# Corrigendum: Achieving inter- and transdisciplinarity in Ecohealth: insights from a rodent-borne disease project in a polycrisis era

**DOI:** 10.3389/fvets.2025.1560103

**Published:** 2025-01-29

**Authors:** Isabelle Arpin, Clémence Massart, Vincent Bourret, Guillaume Castel, Valeria Carolina Colombo, Jana Eccard, Jasmin Firozpoor, Maciej Grzybek, Heikki A. Henttonen, Herwig Leirs, Andrew McManus, Ben Roche, Tarja Sironen, Vincent Sluydts, Peter Stuart, Annetta Zintl, Nathalie Charbonnel

**Affiliations:** ^1^Institut national de recherche pour l'agriculture, l'alimentation et l'environnement (INRAE), Grenoble, France; ^2^Comportement et Ecologie de la Faune Sauvage (CEFS), Institut national de recherche pour l'agriculture, l'alimentation et l'environnement (INRAE), Toulouse, France; ^3^Centre de Biologie pour la Gestion des Populations (CBGP), Institut national de recherche pour l'agriculture, l'alimentation et l'environnement (INRAE), Montpellier, France; ^4^University of Antwerp, Antwerp, Belgium; ^5^University of Potsdam, Potsdam, Germany; ^6^Medical University of Gdańsk, Gdansk, Poland; ^7^Natural Resources Institute Finland (Luke), Helsinki, Finland; ^8^Munster Technological University, Cork, Ireland; ^9^UMR5290 Maladies Infectieuses et Vecteurs: Ecologie, Génétique, Evolution et Contrôle (MIVEGEC), Montpellier, France; ^10^University of Helsinki, Helsinki, Finland; ^11^University College Dublin, Dublin, Ireland

**Keywords:** wicked problems, wickedness, interdisciplinary research, transdisciplinary research, Ecohealth, rodent-borne diseases, polycrisis era

In the published article, there were several errors in the article affiliations. In addition to a correction to the order of affiliations, the following additional affiliations have been added:

^“2^Comportement et Ecologie de la Faune Sauvage (CEFS), Institut national de recherche pour l'agriculture, l'alimentation et l'environnement (INRAE), Toulouse, France; ^3^Centre de Biologie pour la Gestion des Populations (CBGP), Institut national de recherche pour l'agriculture, l'alimentation et l'environnement (INRAE), Montpellier, France.”

In the published article, there was an error in affiliation 1. Instead of “Institut national de recherche pour l'agriculture, l'alimentation et l'environnement (INRAE), Paris, France” it should be “Institut national de recherche pour l'agriculture, l'alimentation et l'environnement (INRAE), Grenoble, France”.

In the published article, there was an error in affiliation 6, previously affiliation 4. Instead of “University of Gdansk, Gdansk, Poland” it should be “Medical University of Gdańsk, Gdansk, Poland”.

The corrected affiliations list appears below:

Isabelle Arpin^1*^, Clémence Massart^1^, Vincent Bourret^2^, Guillaume Castel^3^, Valeria Carolina Colombo^4^, Jana Eccard^5^, Jasmin Firozpoor^5^, Maciej Grzybek^6^, Heikki A. Henttonen^7^, Herwig Leirs^4^, Andrew McManus^8^, Ben Roche^9^, Tarja Sironen^10^, Vincent Sluydts^4^, Peter Stuart^8^, Annetta Zintl^11^ and Nathalie Charbonnel^3^

^1^Institut national de recherche pour l'agriculture, l'alimentation et l'environnement (INRAE), Grenoble, France

^2^Comportement et Ecologie de la Faune Sauvage (CEFS), Institut national de recherche pour l'agriculture, l'alimentation et l'environnement (INRAE), Toulouse, France

^3^Centre de Biologie pour la Gestion des Populations (CBGP), Institut national de recherche pour l'agriculture, l'alimentation et l'environnement (INRAE), Montpellier, France

^4^University of Antwerp, Antwerp, Belgium

^5^University of Potsdam, Potsdam, Germany

^6^Medical University of Gdańsk, Gdansk, Poland

^7^Natural Resources Institute Finland (Luke), Helsinki, Finland

^8^Munster Technological University, Cork, Ireland

^9^UMR5290 Maladies Infectieuses et Vecteurs: Ecologie, Génétique, Evolution et Contrôle (MIVEGEC), Montpellier, France

^10^University of Helsinki, Helsinki, Finland

^11^University College Dublin, Dublin, Ireland

The authors apologize for this error and state that this does not change the scientific conclusions of the article in any way. The original article has been updated.

